# CD73 Expressed on γδ T Cells Shapes Their Regulatory Effect in Experimental Autoimmune Uveitis

**DOI:** 10.1371/journal.pone.0150078

**Published:** 2016-02-26

**Authors:** Dongchun Liang, Aijun Zuo, Ronglan Zhao, Hui Shao, Willi K. Born, Rebecca L. O'Brien, Henry J. Kaplan, Deming Sun

**Affiliations:** 1 Doheny Eye Institute and Department of Ophthalmology, David Geffen School of Medicine at UCLA, Los Angeles, California 90033, United States of America; 2 Department of Ophthalmology and Visual Sciences, Kentucky Lions Eye Center, University of Louisville, Louisville, Kentucky 40202, United States of America; 3 Department of Biomedical Research, National Jewish Health, Denver, Colorado 80206, United States of America; 4 Department of Medical Laboratory, Key Laboratory of Clinical Laboratory Diagnostics in University of Shandong, Weifang Medical University, Weifang 261053, Shandong, China; Ohio State University, UNITED STATES

## Abstract

γδ T cells can either enhance or inhibit an adaptive immune response, but the mechanisms involved are not fully understood. Given that CD73 is the main enzyme responsible for conversion of AMP into the immunosuppressive molecule adenosine, we investigated its role in the regulatory function of γδ T cells in experimental autoimmune uveitis (EAU). We found that γδ T cells expressed different amounts of CD73 during the different stages of EAU and that low CD73 expression on γδ T cells correlated with enhanced Th17 response-promoting activity. Functional comparison of CD73-deficient and wild-type B6 (CD73^+/+^) mice showed that failure to express CD73 decreased both the enhancing and suppressive effects of γδ T cells on EAU. We also demonstrated that γδ T cells expressed different amounts of CD73 when activated by different pathways, which enabled them to either enhance or inhibit an adaptive immune response. Our results demonstrate that targeting CD73 expression on γδ T cells may allow us to manipulate their pro- or anti-inflammatory effect on Th17 responses.

## Introduction

Multiple lines of evidence demonstrate that γδ T cells have a strong regulatory effect on immune responses [1,2], but the mechanisms involved remain unclear. We have previously reported that regulation of the Th17 response by γδ T cells in a mouse model of human uveitis, experimental autoimmune uveitis (EAU), is determined by their activation status, with activated γδ T cells enhancing Th17 autoimmune responses and non-activated cells being either non-functional or suppressive [3–6]. Knowledge of how activation affects the pro- and anti-inflammatory activity of γδ T cells and how γδ T cells are activated in different pathogenic processes should provide clues about the pathogenic mechanism of autoimmune diseases, particularly Th17 autoimmune responses. In a previous report, we demonstrated that, depending on their activation status and level of expression of the interleukin-23 receptor (IL-23R), mouse γδ T cells can either enhance or inhibit the Th17 autoimmune responses in EAU [4].

The purinergic system is an evolutionally selected system modulating immune functions [7,8]. Release of adenosine triphosphate (ATP) into the extracellular space is elicited by tissue damage, such as that caused by inflammation. Under physiological conditions, ATP is present exclusively within cells, but stimulation of almost all mammalian cell types leads to its release [8]. Once released into the extracellular space, ATP is hydrolyzed in a stepwise manner into adenosine diphosphate (ADP), adenosine-5iphosphate (ADP)ce, A, and finally, adenosine by ectonucleotidases, including CD73 and CD39 [9]. Cells that express CD39 and CD73 may act to suppress inflammatory responses through the production of adenosine [10,11]. While ATP acts on many immune cells to promote inflammation [12–15], the action of ATP metabolites, especially adenosine, is mainly anti-inflammatory [7,8]. Multiple lines of evidence show that binding of adenosine to its receptors modulates the outcome of various pathophysiological conditions, including autoimmune diseases and cancers [16–18]. Thus, assessing the extent of the degradation of ATP to adenosine in immune-related diseases should assist in determining the balance of pro- and anti-inflammatory effects in the pathogenesis of diseases.

CD73 is the main enzyme responsible for the conversion of AMP into immunosuppressive adenosine [19–23]. We have previously shown that CD73 expressed on γδ T cells is highly active in the conversion of AMP to adenosine and that activated γδ T cells express lower levels of CD73 than naïve cells [3,17]. In the present study, we examined whether CD73 expression is important in the regulatory function of γδ T cells by comparing γδ T cells isolated from CD73-deficient (CD73^-/-^) and wild-type (WT) B6 (CD73^+/+^) mice. γδ T cells were found to express different amounts of CD73 during different disease phases. We showed that the level of CD73 expression correlated with the pro- and anti-inflammatory activities of γδ T cells in the regulation of Th17 autoimmune responses in EAU. These results suggest that it may be possible to modulate Th17 autoimmune responses by manipulating CD73 expression on γδ T cells.

## Materials and Methods

### Animals and reagents

Female C57BL/6 (B6), IFN-γ^-/-^, CD73^-/-^, and T cell receptor (TCR)-δ^-/-^ mice on the B6 background were purchased from Jackson Laboratory (Bar Harbor, ME), and TCR-δ^-/-^IFN-γ^-/-^ double knockout mice were bred in our own colony; 8- to 16-week-old mice were used in all studies. The mice were housed and maintained in the animal facilities of the University of California Los Angeles. All animal studies conformed to the Association for Research in Vision and Ophthalmology Statement for the Use of Animals in Ophthalmic and Visual Research. Institutional approval was obtained from the Institutional Animal Care and Use Committee of the Doheny Eye Institute, University of California Los Angeles, and institutional guidelines regarding animal experimentation were followed.

Recombinant murine IL-12 and IL-23 were purchased from R & D Systems (Minneapolis, MN). Fluorescein isothiocyanate (FITC)- or phycoerythrin (PE)-conjugated antibodies against the mouse αβ TCR, γδ TCR, IL-17, IFNγ, CD3, CD73, CD44, and CD4 and isotype control antibodies were purchased from e-Bioscience (San Diego, CA). AMP and the CD73 inhibitor α,β-methylene ADP (APCP) were purchased from Sigma-Aldrich (St. Louis, MO).

### Induction of EAU

Active EAU was induced in described mouse strains by subcutaneous injection on day 0 of an emulsion containing 200 μg of the human interphotoreceptor retinoid-binding protein (IRBP) peptide IRBP_1-20_ (Sigma-Aldrich) in complete Freund’s adjuvant (CFA) (Difco, Detroit, MI) at six spots at the tail base and on the flank, and intraperitoneal (i.p.) injection of 300 ng of pertussis toxin, after anesthetized with a combination of ketamine and xylazine [4,5,24].

The mice were examined three times a week for clinical signs of EAU by indirect fundoscopy, in which the pupils were dilated using 0.5% tropicamide and 1.25% phenylephrine hydrochloride ophthalmic solutions and fundoscopic grading of disease was performed using the scoring system reported previously [25]. Histopathological evaluation was performed on eye sections at the end of the experiment, and disease was graded pathologically based on cellular infiltration and structural changes as described previously [26,27]. In some studies, 2 x 10^6^ γδ T cells isolated from IRBP_1-20_ immunized B6 (CD73^+/+^) or CD73^-/-^ mice on day 13 post-immunization were injected i.p into recipient mice just before they were injected with IRBP_1-20_/CFA.

### Cell preparation

For examination of cell function, the immunized mice were euthanized 13 days after immunization by exposure with an overdose of sodium pentobarbital. αβ T cells were purified from the spleens or draining lymph nodes of IRBP_1-20_-immunized TCR-δ^-/-^ mice and γδ T cells from immunized B6 mice, by positive selection using a combination of FITC-conjugated anti-CD3 antibody and anti-FITC antibody-coated Microbeads, followed by separation using an auto-MACS separator system according to the manufacturer’s suggested protocol (Miltenyi Biotec, Auburn, CA). The purity of the isolated cells, determined by flow cytometric analysis using PE-conjugated antibodies against αβ or γδ T cells, was >95%.

### Generation of bone marrow dendritic cells

Bone marrow dendritic cells (BMDCs) were generated by incubation of bone marrow cells from B6 mice for 5 days in the presence of 10 ng/ml of recombinant murine granulocyte macrophage colony-stimulating factor (R&D Systems), as described previously [28]. To test the stimulating effect of these cells on γδ T cells, the BMDCs were pre-treated for 48 h with 100 ng/ml of LPS [29].

γδ T cells were separated from either naïve B6 mice or IRBP_1-20_-immunized B6 mice, as indicated in the text, by positive selection using a combination of FITC-conjugated anti-TCR-δ antibody and anti-FITC antibody-coated Microbeads, followed by separation using an auto-MACS. To test the effect of activation by cytokines or DCs, freshly prepared γδ T cells from IRBP_1-20_-immunized B6 mice were cultured in cytokine-free medium for 5 days to generate the resting state, as γδ T cells freshly isolated from IRBP_1-20_-immunized mice are activated; the cells were then incubated for 48 h with a combination of IL-1, IL-7, and IL-23 (10 ng/ml of each) as described previously [4] or for 48 h with LPS-activated DCs [29] at a γδ T cell:DC ratio of 10:1.

### Measurement of the in vitro enhancing and inhibitory effects of γδ T cells on Th1 and Th17 responses

CD3^+^ T cells (1 x 10^6^) from IRBP_1-20_-immunized TCR-δ^-/-^ mice on day 13 post-immunization were cultured at 37°C in 24-well plates under either Th1 polarizing conditions (culture medium supplemented with 10 ng/ml of IL-12) or Th17 polarizing conditions (culture medium supplemented with 10 ng/ml of IL-23) in a total volume of 600 μl. The percentage of IFN-γ^+^ and IL-17^+^ T cells among the responder T cells was determined as described previously after 5 days of culture [3,4,17] and cytokine levels in the culture supernatants were measured after 48 h of culture as described below. When the functions of γδ T cells activated by different methods and γδ T cells of different genotypes were compared, 2 x 10^4^ γδ T cells (5% of the total T cells) were included in the cultures.

### Cell staining and immunofluorescence flow cytometry

In vivo primed T cells were incubated for 5 days with the immunizing antigen and autologous irradiated spleen cells as antigen-presenting cells (APCs), then T cells were separated using Ficoll gradient centrifugation and stimulated in vitro for 4 h with 50 ng/ml of phorbol myristic acetate, 1 μg/ml of ionomycin, and 1 μg/ml of brefeldin A (all from Sigma, St. Louis, MO). Aliquots of 2 x 10^5^ cells were then surface-stained and/or intracellularly stained with combinations of FITC- or PE-conjugated monoclonal antibodies, as described previously [4,17,30]. Data collection and analysis were performed on a FACS_calibur_ flow cytometer using CellQuest software.

### ELISA measurement of cytokine levels in serum and culture supernatants

ELISA kits (E-Bioscience) were used to measure serum IFN-γ and IL-17 levels on day 13 post-immunization and in the 48 h culture supernatants of responder T cells isolated on day 13 post-immunization from IRBP_1-20_-immunized B6 or TCR-γ^-/-^ mice.

### CFSE and thymidine incorporation proliferation assays

Purified CD3^+^ T cells from IRBP_1-20_-immunized TCR-δ^-/-^ mice were stained with CFSE (Sigma-Aldrich) as described previously [31]. Briefly, the cells were washed and suspended at 50 x 10^6^ cells/ml in serum-free RPMI 1640 medium; cells were then incubated at 37°C for 10 min with gentle shaking with a final concentration of 5 μM CFSE before being washed twice with, and suspended in, complete medium, stimulated with immunizing peptide in the presence of APCs, and analyzed by flow cytometry.

To assay thymidine incorporation, T cells from IRBP _1-20_-immunized TCR-δ-/- mice were seeded at 4 x 10^5^ cells/well in 96-well plates, then cultured at 37°C for 48 h in a total volume of 200 μl medium with IRBP_1-20_ and APCs, with or without addition of 5% γδ T cells from B6 (CD73+/+) or CD73-/- mice; [3H] thymidine incorporation during the last 8 h was assessed using a microplate scintillation counter. The proliferative response was expressed as the mean cpm ± standard deviation (SD) of triplicate determinations.

### HPLC assay of AMP conversion to adenosine by CD73

αβ or γδ T cells were washed in Hanks’ balanced salt solution (HBSS) and suspended in HBSS at 1x10^6^ cells/ml, and 100 μl of the suspension was incubated for 1 h at 37°c with 1 mM AMP. The cells were then spun down and the supernatants diluted 10-fold with time-division multiplexing buffer (100 mM Tris, pH 7.4, 0.8 mM MgCl_2_, 1 mM mercaptoethanol) and analyzed for adenosine by HPLC. A reverse-phase HPLC column (Agilent Technologies, Santa Clara, CA, C18, particle size 5 μm, 250×4.6 mm) and a linear gradient of 0–50% methanol in water (1 ml/min) were used; and the absorbance of the eluate was monitored at 260 nm. The area under the adenosine peak was calculated using a computer program (Millennium Software), and the concentration of adenosine in the sample was obtained by reference to a standard curve for adenosine. In studies that used the CD73 inhibitor APCP, 3 μM APCP was included in the medium throughout the test.

### Statistical analysis

The results in the figures are from a representative experiment, which was repeated 3–5 times. The statistical significance of differences between groups in a single experiment was initially analyzed by ANOVA, and, if a statistical significance was detected, the Student–Newman–Keuls post-hoc test was subsequently used. A *P* value less than 0.01 was considered statistically significant (indicated as **).

## Results

### CD73 expression is downregulated on γδ T cells during the pre-clinical phase of EAU and is restored during the clinical phase

**Fig 1:**
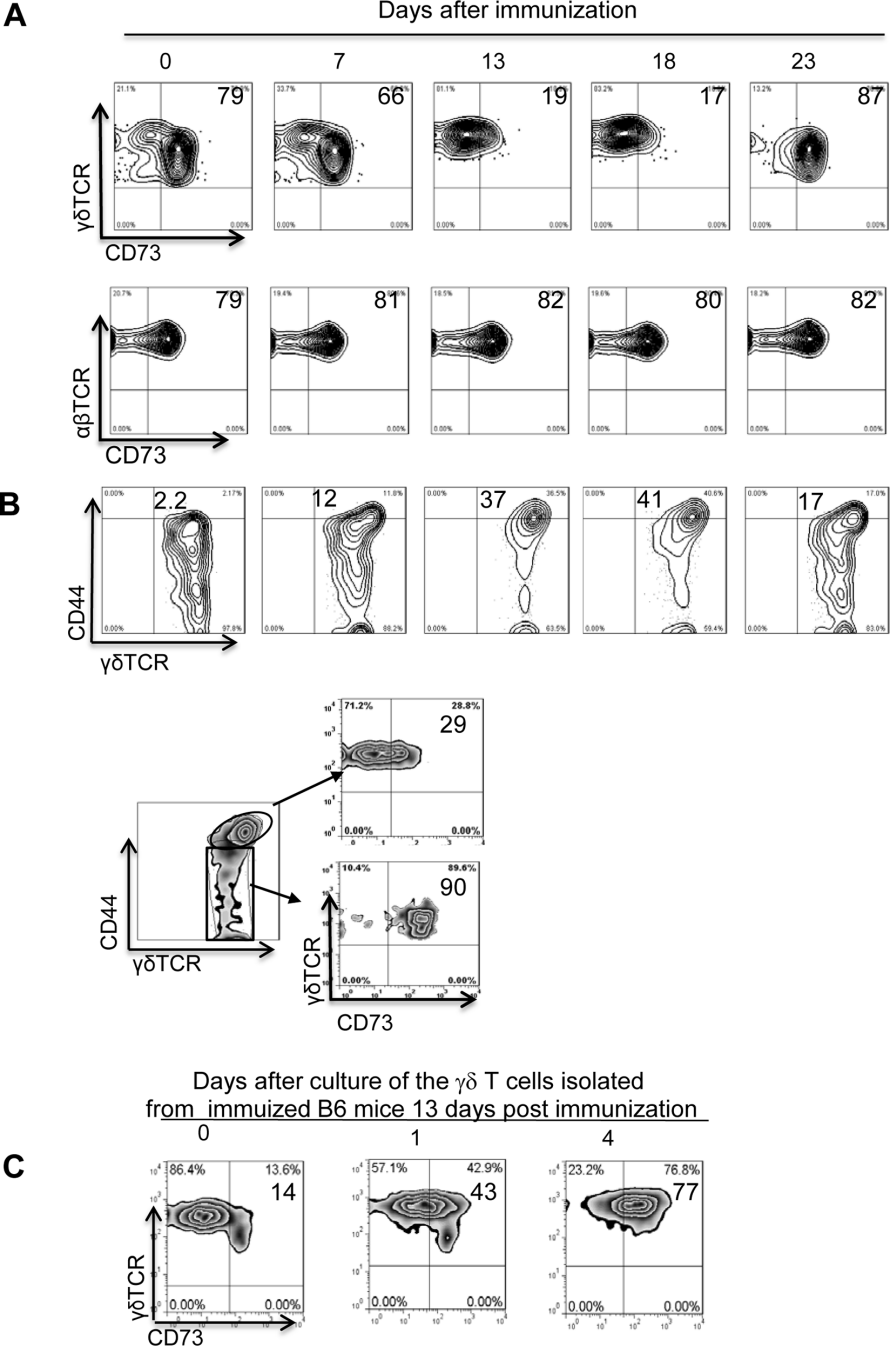
CD73 expression by γδ T cells is downregulated and CD44 expression upregulated during the pre-clinical phase of EAU. Splenic T cells from IRBP_1-20_-immunized B6 mice on the indicated day post-immunization were double- stained with anti-mouse CD73 and anti-mouse γδTCR antibodies (upper panels) or anti-mouse CD73 and anti-mouse αβ antibodies (lower panels). Expression of CD73 by αβ and γδ T cells was assessed from gated γδTCR^+^ or αβTCR^+^ populations. CD44^high^ γδ T cells express low levels of CD73, whereas CD44^low^ γδ T cells express high levels of CD73. CD3^+^ spleen cells were isolated from IRBP_1-20_-immunized mice at day 13 post-immunization, and gated CD44^high^ and CD44^low^ γδ T cells were analysed for expression of CD73. γδ T cells freshly prepared from IRBP_1-20_-immunized mice (day 13) were cultured in cytokine-free medium for 1 or 4 days, and a fraction of the cells was double-stained with anti-mouse γδ TCR and anti-mouse CD73 antibodies. The results in (A-C) are for a single experiment and are representative of those obtained in three studies.

We have previously shown that γδ T cells in mice with induced EAU are activated during the pre-clinical stage and that this is associated with their ability to promote Th17 responses [4–6,24]. To determine the mechanisms by which γδ T cell activation leads to augmented autoimmune responses, we investigated the role of CD73. Previously, we showed that CD73 expressed on γδ T cells can convert AMP to adenosine [3]. In the present study, a kinetic study performed on B6 mice immunized with IRBP_1-20_ on day 0 showed that, at days 7–18 post-immunization (shortly before clinical expression of EAU begins at 18–20 days post-immunization), splenic γδ T cells expressed significantly decreased levels of CD73 compared to γδT cells from naïve mice (day 0 before immunization) (Fig 1A, upper panels). Almost 80% of splenic γδ T cells expressed CD73 before immunization; this percentage dropped to 19% on day 13 and 17% by day 18 post-immunization and returned to high levels (87%) by day 23. Parallel examination of αβ T cells (Fig 1A, lower panels) showed that CD73 expression on αβ T cells remained unchanged throughout the test period. The top panels of Fig 1B show that the decreased expression of CD73 was accompanied by increased CD44 expression on γδ T cells and the lower panels show that, on day 13, the CD44^high^ γδ T cells were CD73^low^ and the CD44^low^ cells were CD73^high^. In addition, γδ T cells freshly isolated from IRBP_1-20_-immunized B6 mice on day 13 post-immunization expressed low levels of CD73; but after 5 days of culture in cytokine-free medium, levels increased to those seen in naïve mice (Fig 1C), suggesting that CD73 on γδ T cells is transiently downregulated when the cells become activated.

### Activated CD73^-/-^ γδ T cells have greater Th17 response-promoting activity in vivo and in vitro

**Fig 2:**
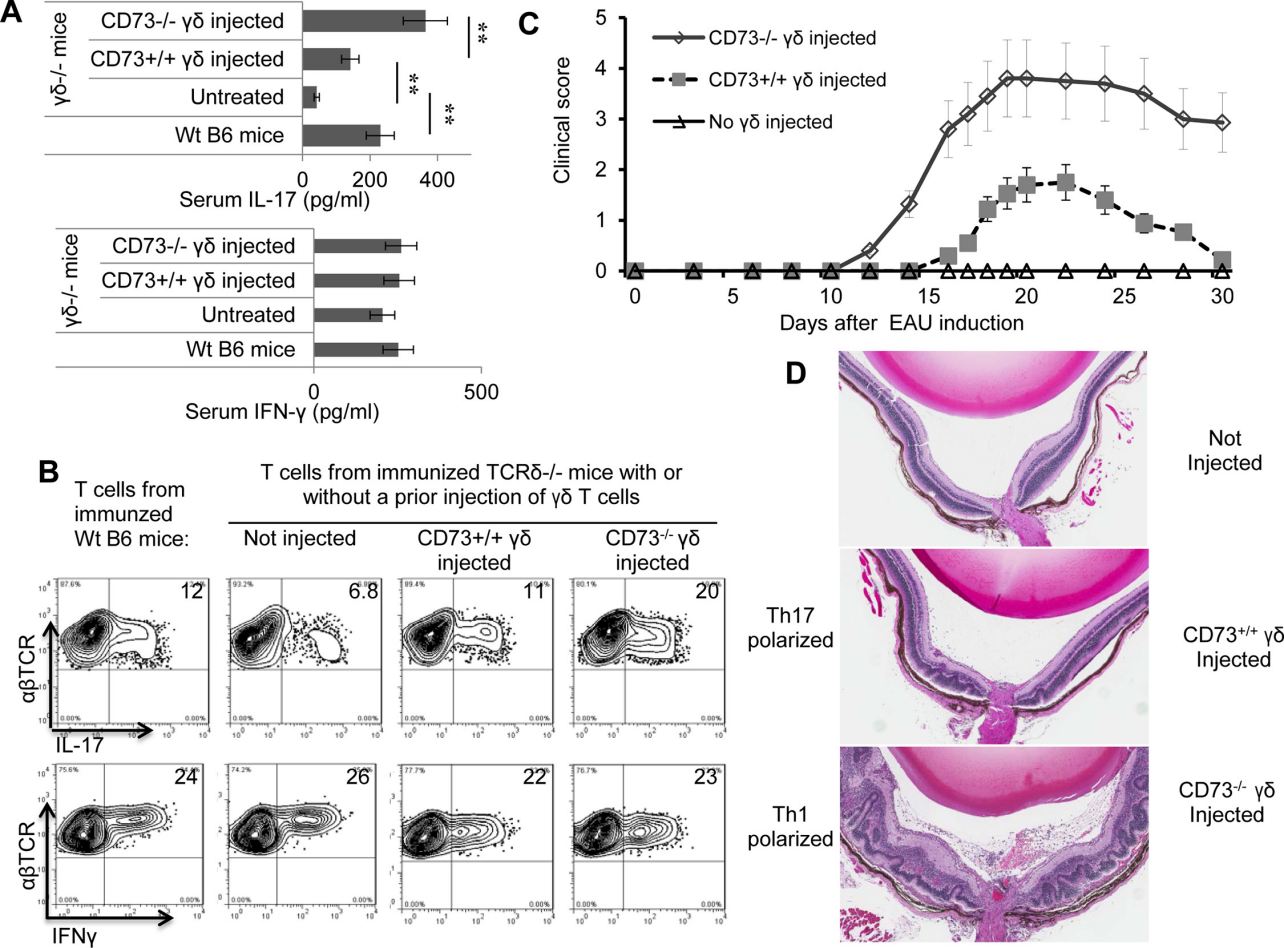
CD73^-/-^ γδ T cells have a greater proinflammatory effect than CD73^+/+^ γδ T cells. A-B) γδ T cells isolated from IRBP_1-20_-immunized B6 (CD73^+/+^) or CD73^-/-^ mice on day 13 post-immunization were injected (1 x 10^6^/recipient) into TCR-δ^-/-^ recipients. The recipient mice were then immunized with IRBP_1-20_/CFA, and tests were performed on day 13 post-immunization. (A) shows serum levels of IL-17 (upper panels) and IFN-γ (lower panels) in the recipients. (B) CD3+ T cells were isolated and stimulated for 5 days with the immunizing peptide and APCs under either Th17 (top panels) or Th1 (lower panels) polarizing conditions; then intracellular staining for IL-17^+^ and IFN-γ^+^ cells among the TCRαβ^+^ responder T cells was assessed. In both (A) and (B) controls included immunized TCR-δ^-/-^ and B6 mice that were not injected with γδ T cells. (C and D) Three groups of TCR-δ^-/-^IFN-γ^-/-^ double KO mice (n = 6) were immunized with IRBP_1-20_ 300 μg/CFA on day 0 with or without prior injection of γδ T cells (1 x 10^6^/recipient) isolated from IRBP_1-20_-immunized B6 (CD73^+/+^) or CD73^-/-^ mice on day 13 post-immunization. (C) shows the time course of disease over 30 days measured by fundoscopy starting 10 days post immunization. (D) On day 25 post-immunization, the mice were sacrificed and the eyes taken and subjected to pathological examination. The panels shows H&E staining of an eye section from TCR-δ^-/-^ recipients that received no injected γδ T cells and ones injected with CD73^+/+^ or CD73^-/-^ γδ T cells. The data are from one single experiment and are representative of those obtained in three independent experiments. **p< 0.01.

Our previous studies demonstrated that injection of a small percentage of γδ T cells from IRBP _1-20_-immunized B6 mice makes it possible to induce EAU in recipient TCR-δ^-/-^ mice, which are normally non-susceptible to induction of EAU [4,5,32]. To determine whether CD73^+/+^ and CD73^-/-^ γδ T cells have different abilities to increase Th1 or Th17 responses in vivo, we injected 4 sets of mice (B6 mice, untreated TCR-δ^-/-^ mice, and TCR-δ^-/-^ mice that had undergone adoptive transfer of γδ T cells from IRBP_1-20_-immunized CD73^+/+^ or CD73^-/-^ mice) with IRBP_1-20_/CFA and analyzed serum cytokine levels and the Th1 and Th17 responses of the recipients at day 13 post-immunization (the time point at which the greatest T cell changes are seen) [4,32,33]. Fig 2A shows that serum IL-17 levels were significantly increased in TCR-δ^-/-^ mice injected with either type of γδ T cell and that the increase was greater in the CD73^-/-^ γδ T cell recipients, while serum IFN-γ levels were essentially the same in all 4 groups. Fig 2B shows that, when T cells purified from the spleens and draining lymph nodes were subjected to in vitro stimulation with the immunizing peptide and APCs under culture conditions favoring either Th1 autoreactive T cell expansion (IL-12-containing medium, top panels) or Th17 autoreactive T cell expansion (IL-23-containing medium, bottom panels) and the T cells separated by Ficoll gradient centrifugation and stained intracellularly with FITC-labeled anti–IL-17 or anti-IFNγ antibodies, increased IL-17 expression was seen in both sets of cells from mice that received γδ T cells; but again, a greater difference was seen in recipients of CD73^-/-^ γδ T cells (top panels), whereas no difference was seen in the percentage of IFNγ-stained cells (bottom panels).

We also injected γδ T cells from IRBP_1-20_-immunized B6 mice (CD73^+/+^) or CD73^-/-^ mice into TCR-δ^-/-^ or TCR-δ^-/-^IFN-γ^-/-^ recipient mice, with a non-injected group as controls, and examined susceptibility to induction of EAU by immunization with a pathogenic dose of IRBP _1–20._ We found that both the TCR-δ^-/-^ and TCR-δ^-/-^IFN-γ^-/-^ recipient mice gained increased EAU susceptibility after γδ T cell injection, with a greater effect in the TCR-δ^-/-^IFN-γ^-/-^ recipients (not shown). Fig 2C shows time-course results and Fig 2D shows eye pathology results for the TCR-δ^-/-^IFN-γ^-/-^ mice and demonstrates that, without γδ T cell injection, the mice were not susceptible to induction of EAU, whereas, after injection of CD73^+/+^ γδ T cells from IRBP_1-20_-immunized B6 mice, EAU susceptibility was restored, and an even greater effect was seen using γδ T cells from IRBP_1-20_-immunized CD73^-/-^ mice.

### CD73^+/+^ γδ T cells, but not CD73^-/-^ γδ T cells, have an inhibitory effect on the proliferation of CSFE-labeled αβ T cells in the presence of AMP

**Fig 3:**
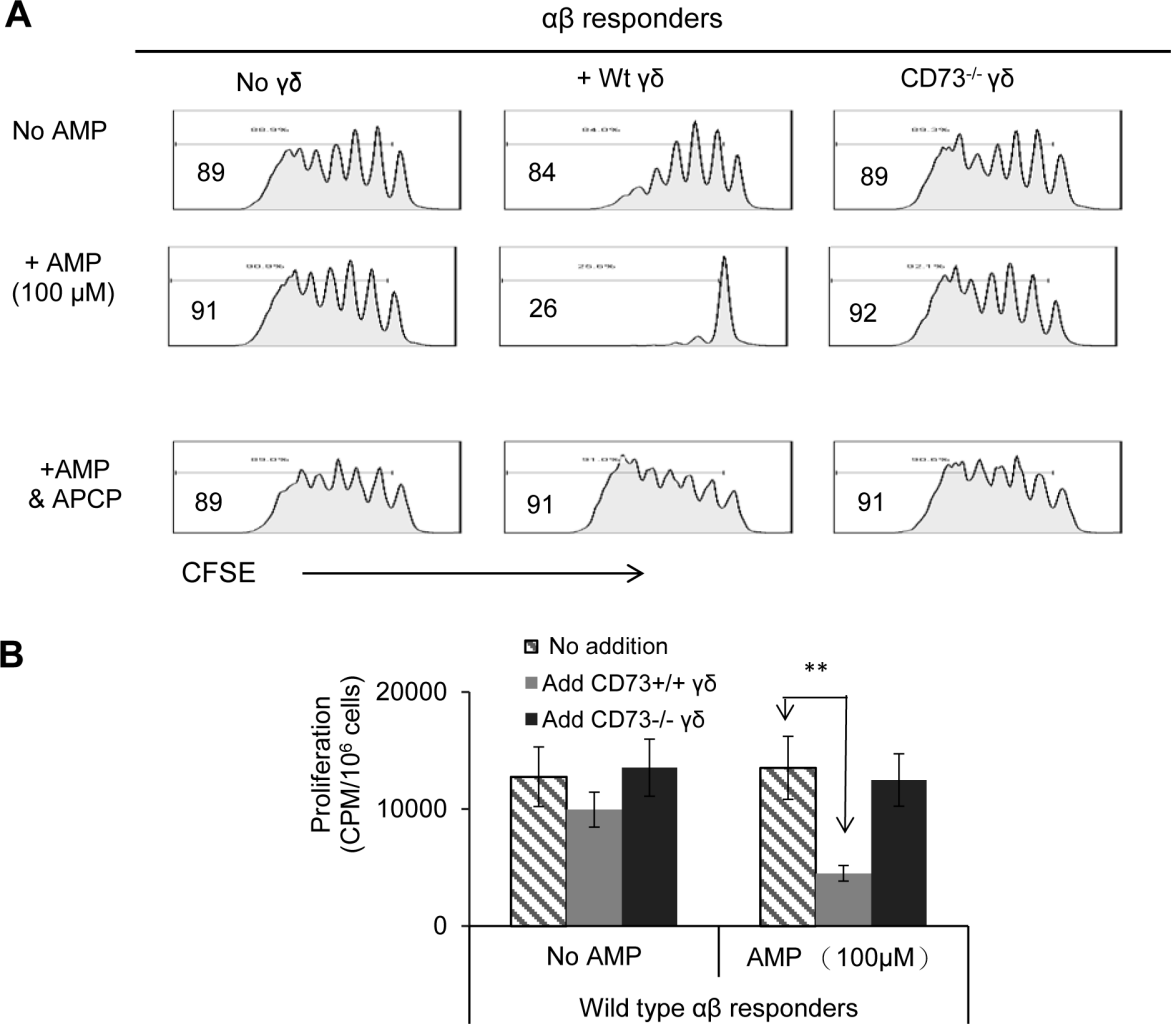
AMP only inhibits the proliferation of αβ T cells in the presence of CD73^+/+^ γδ T cells. A) CSFE-labelled CD3^+^ T cells purified from IRBP_1-20_-immunized TCR-δ^-/-^ mice on day 13 post-immunization were stimulated for 5 days with the immunizing peptide and APCs in the absence (top panels) or presence (center panels) of AMP (100 μM) with or without addition of γδ T cells from IRBP_1-20_-immunized CD73^-/-^ or CD73^+/+^ mice (5% of total responder T cells). T cells were then separated by Ficoll gradient centrifugations and subjected to FACS analysis. B) Thymidine incorporation proliferation assay of the responder T cells described in (A).

AMP is one of the metabolites of ATP. Our previous results showed that AMP failed to inhibit the proliferation of CSFE-labeled αβ T cells in the absence of γδ T cells. But AMP was inhibitory when the responder T cells contained a small percentage (5%) of γδ T cells from IRBP_1-20_-immunized CD73^+/+^ mice, and this inhibitory effect was prevented by the CD73 inhibitor APCP [3], suggesting that the CD73 molecules on γδ T cells convert the non-suppressive AMP into suppressive adenosine. To test whether γδ T cells from IRBP_1-20_-immunized CD73^-/-^ mice were capable of suppression, AMP was added to CSFE-labeled responder T cells from IRBP_1-20_-immunized TCR-δ^-/-^ mice in the presence or absence of γδ T cells from IRBP_1-20_-immunized CD73^-/-^ or CD73^+/+^ mice. As shown by a FACS study in Fig 3A and by thymidine incorporation in Fig 3B, AMP-induced inhibition of proliferation was seen only in the presence of CD73^+/+^ γδ T cells; and as shown in Fig 3A, this effect was blocked by APCP.

### Cytokine-activated γδ T cells express high CD73 levels, whereas DC-activated γδ T cells express low levels

**Fig 4:**
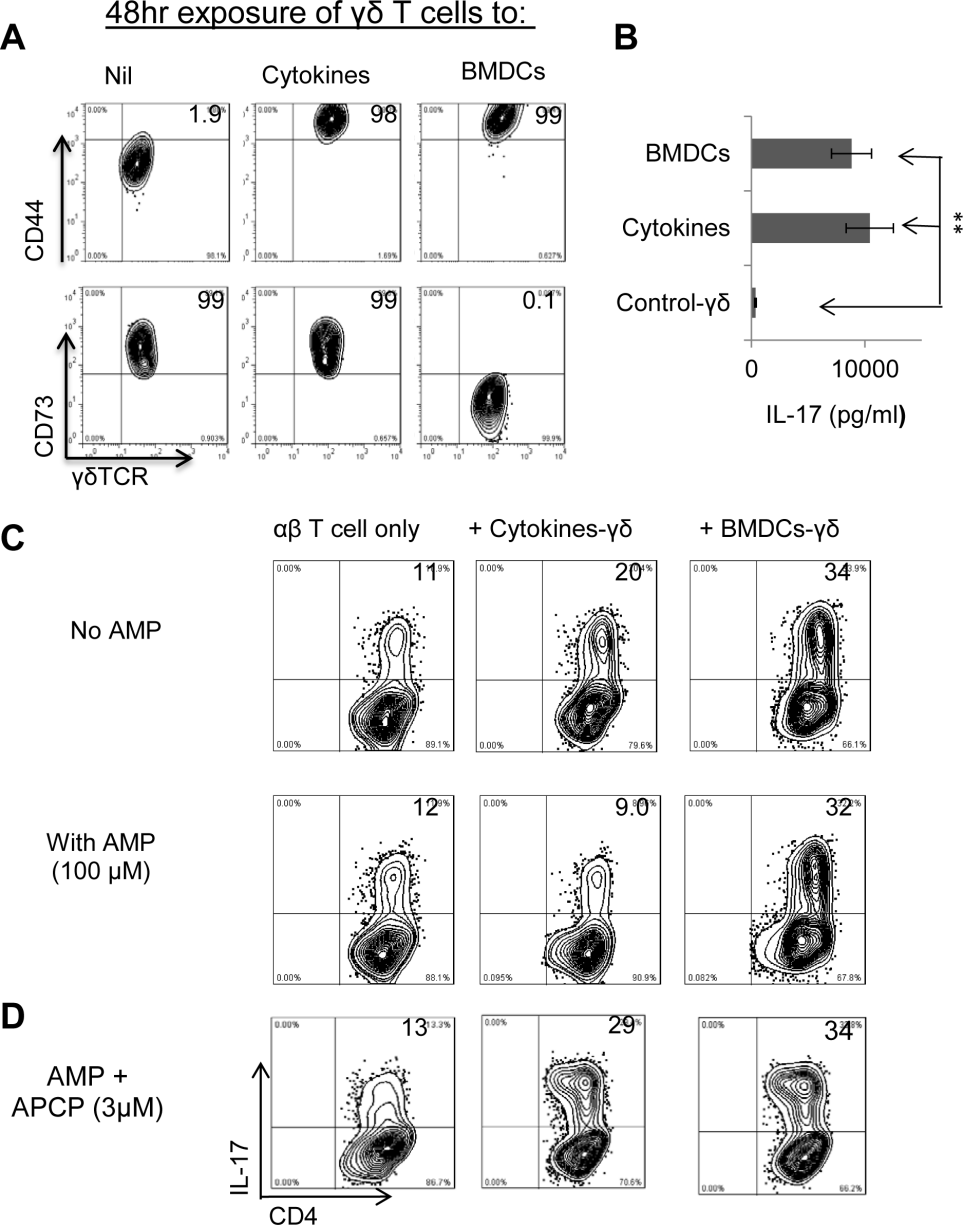
Cytokine-activated γδ T cells are CD73^high^, whereas DC-activated γδ T cells are CD73^low^. The γδ T cells were isolated from B6 mice 13 days post immunization. Before further test, they were cultured in cytokine-free medium for at least 4 days to acquire resting status (see Fig 1C). A-B) Aliquots of γδ T cells purified from IRBP_1-20_-immunized B6 mice on day 13 post-immunization were cultured for 48 h in cytokine-free medium (Nil) or with the cytokine mixture (10 ng/ml each of IL-1, IL-7, and IL-23 (cytokines) or with LPS-activated BMDCs. (A) The γδ T cells were then separated and double-stained with anti-TCR-δ and anti-CD44 antibodies (upper panels) or anti-TCR-δ and anti-CD73 antibodies (lower panels), and (B) the supernatants were assayed for IL-17 by ELISA. C). Test of the ability of the two types of activated γδ T cells to inhibit αβ T cell function in the presence of AMP. Aliquots of responder αβ T cells prepared from IRBP_1-20_-immunized TCR-δ^-/-^ mice on day 13 post immunization (1 x 10^6^/well) were stimulated for 5 days in 24-well plates with the immunizing peptide and splenic APCs, in the absence (left panels) or presence of cytokine-activated (center panels) or DC-activated (right panels) γδ T cells from IRBP_1-20_-immunized B6 mice (5% of total responder T cells) and in the absence (upper panels) or presence (lower panels) of 100 μM AMP. Proliferating cells were then measured by FACS analysis after cytoplasmic staining with anti-IL-17 antibodies and surface staining with anti-CD4 antibodies. D) Test of the effect of the CD73 inhibitor APCP. The studies described in (C) were repeated in the absence or presence of 3 μM APCP throughout the test.

Previous studies have shown that γδ T cells can be activated in the absence of TCR ligation [34–37]. A combination of the cytokines IL-1, IL-7, and IL-23 [5,34,35], various TLR ligands [5], or dendritic cells (DCs) previously incubated with a TLR ligand [4,30,32] are all strong stimulators of γδ T cells. To examine whether γδ T cells activated via different pathways are functionally distinct, we incubated γδ T cells from IRBP_1-20_-immunized B6 mice with medium, a combination of IL-1, IL-7, and IL-23, or LPS-activated DCs, then examined the degree of activation of the γδ T cells by FACS analysis and cytokine production. Unexpectedly, we found that the cytokine-treated γδ T cells remained highly CD73-positive, while the γδ T cells incubated with DCs became CD73-negative (Fig 4A, lower panels), even though both sets of γδ T cells showed a similar increase in the levels of the T cell activation marker CD44 (Fig 4A, upper panels) and produced comparable amounts of IL-17 (Fig 4B).

We then performed functional comparisons, in which αβ responder T cells purified from IRBP_1-20_-immunized TCR-δ^-/-^ mice were incubated with immunizing antigen and APCs, either alone or in the presence of γδ T cells stimulated by either cytokines or DCs, then were subjected to FACS analysis using anti-IL-17 and anti-CD4 antibodies. As shown in the upper panels of Fig 4C, in the absence of AMP, αβ responder T cells from TCR-δ^-/-^ mice generated a low percentage of IL-17^+^ cells; and the addition of either cytokine-treated or DC-exposed γδ T cells increased the percentage of these cells, with the DC-exposed γδ T cells having a stronger effect. Comparison of the suppressive activity of AMP in the absence or presence of γδ T cells (Fig 4C, bottom panels) showed that the two types of activated γδ T cells were functionally different, as the percentage of IL-17^+^ cells was significantly decreased in cultures containing cytokine-exposed γδ T cells compared to cultures without the γδ T cells, but was increased in cultures containing DC-exposed γδ T cells. This result shows that cytokine-activated γδ T cells, which retain high levels of CD73, are able to exert a suppressive effect in the presence of AMP, while DC-exposed γδ T cells, which have decreased CD73 levels, do not. This idea was further supported by the results of an inhibition test using the CD73 blocker APCP [21,38], which showed that the suppressive effect of cytokine-activated γδ T cells in the presence of AMP was blocked by the inhibitor (Fig 4D).

### CD73 expressed by γδ T cells is more effective in converting AMP to adenosine than CD73 expressed by other immune cells

**Fig 5:**
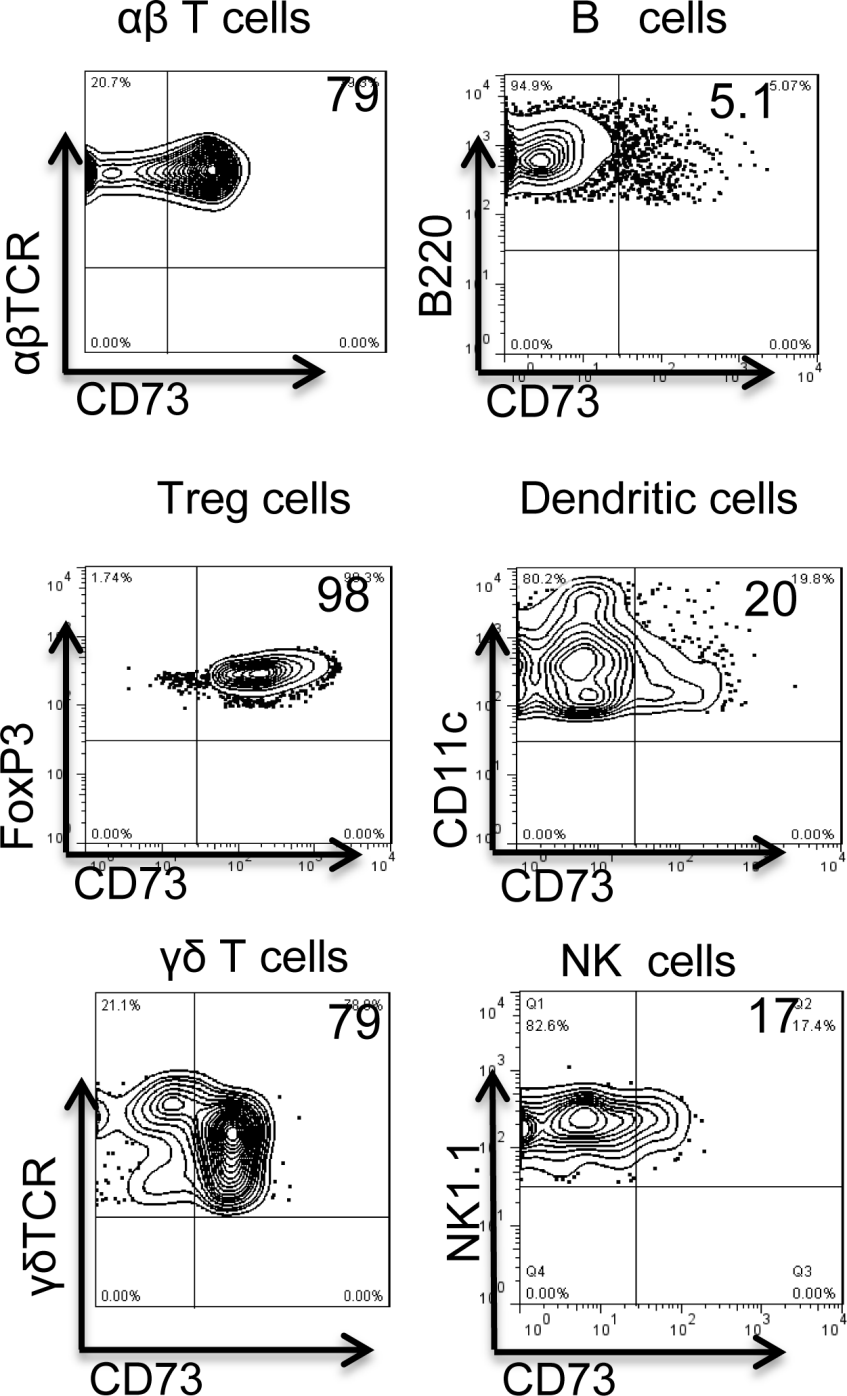
Examination of CD73 expression on, and AMP-degrading activity of, different hematopoietic cells. A) Expression of CD73 by αβ T cells, B cells, Treg cells, DCs, and NK/NKT cells. αβ T cells (αβTCR^+^), B cells (B220^+^), DCs (CD11c^+^), γδT cells (γδTCR^+^), and NK/NKT cells (NK1.1^+^) were purified on a MACS column after staining with the indicated antibodies. Treg cells (Foxp3^+^) cells were prepared in two steps: CD4^+^ T cells were purified from naïve mice, and then CD25^+^ T cells were enriched using kits from Stem Cell Inc.

**Fig 6:**
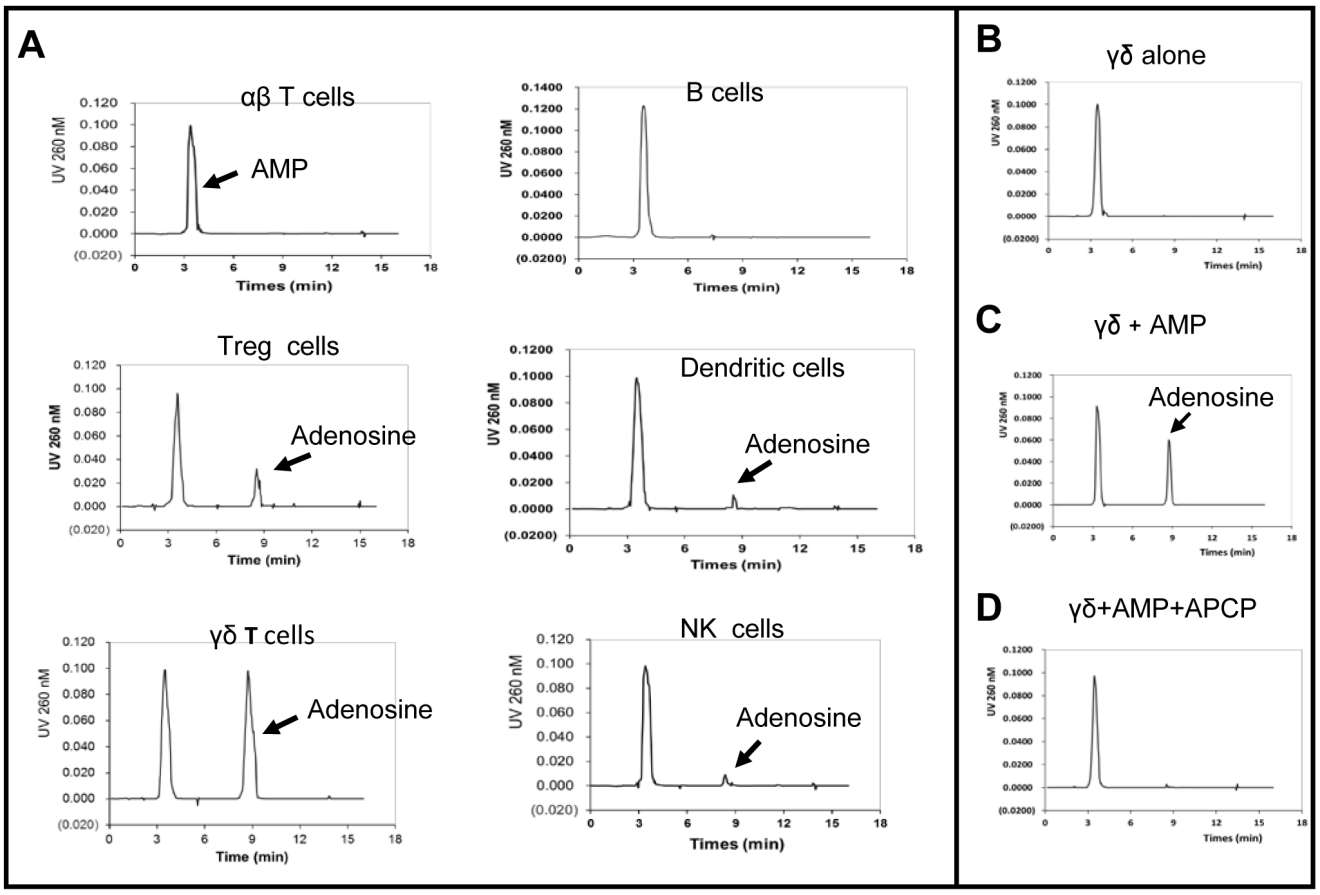
HPLC analysis of the amount of adenosine generated in culture supernatants. A). After 1 h culture of the indicated cells (3 x 10^5^/well) with 1 mM AMP. The adenosine peaks detected in the culture supernatants are indicated by the arrow. The results are for a single experiment and are representative of those obtained in two experiments. B-D) HPLC analysis showing the peaks of adenosine in culture supernatants after 1 h culture of γδ T cells (1 x 10^5^/well) (B), γδ T cells + AMP (C), γδ T cells + AMP + APCP (3 μM) (D) in 96-well plate. Results shown are from a single experiment and are representative of three experiments.

To determine whether CD73 molecules expressed on different immune cells are functionally equal, we compared levels of CD73 expression and the ability to convert AMP to adenosine in αβ T cells, B cells (B220^+^), regulatory T cells (Treg cells; Foxp3^+^), DCs (CD11c^+^), γδ T cells, and NK/NKT cells (NK1.1^+^) from naïve B6 mice. As shown in Fig 5, CD73 was expressed on 5.1% of B cells, 20% of DCs, and 17% of NK cells, but on the majority of Foxp3^+^ T cells (98%), αβ T cells (79%), and γδ T cells (79%). We then compared the ability of these different cells to convert AMP to adenosine using our previously described HPLC assay system [3], in which an equal number of test cells (5 x 10^5^/well) is incubated with 1 mM AMP for 1 h, then the cell-free culture supernatant is examined by HPLC for the presence of adenosine. As shown in Fig 6, γδ T cells were the most effective at converting AMP to adenosine. Foxp3^+^ Treg cells showed modest activity, while minimal activity was seen with NK/NKT cells, B cells, and αβ T cells. The results in Fig 6B–6D show that no adenosine was detectable in the supernatants of γδ T cells cultured in the absence of exogenously added AMP (Fig 6B), while after incubation with 1 mM AMP for 1 h, an adenosine peak (indicated by the arrow) was seen in the γδ T cell cultures (6C); additionally, adenosine generation is almost completely blocked by the addition of a CD73 inhibitor APCP (6D), suggesting that the adenosine is derived from metabolism of exogenously added AMP.

## Discussion

The regulatory effect of γδ T cells on adaptive immunity has been observed repeatedly [39–42]; but how these cells regulate the immune response is poorly understood, and it remains largely obscure how γδ T cells enhance an immune response in some cases but inhibit it in others. It has been noted that the regulatory function of γδ T cells changes as the immune response progresses [43] and upon exposure to environmental factors, such as Toll ligand [44,45] or mycobacteria [46–49]. Our previous studies showed that the regulatory effect of γδ T cells depends on their activation status and that a large proportion of γδ T cells from IRBP_1-20_-immunized B6 mice, but not from naïve B6 mice, are activated [5,25]. An understanding of the factors that affect γδ T cell regulatory activity should facilitate clinical attempts at modulating γδ T cell function to achieve therapeutic goals [50–54]. To better understand the mechanisms by which γδ T cells regulate Th17 responses [3–6,24] and by which activated γδ T cells alter their regulatory effect [3–5], we looked for molecules that cause γδ T cell activation and affect γδ T cell function. In the present study, we found that CD73 molecules expressed on γδ T cells play a major role in modulating their pro- and anti-Th17 response activity, and that CD73 molecules expressed on different hematopoietic cells have different effects on the enzymatic degradation of ATP to adenosine.

Pathological inflammation promotes an accumulation of extracellular ATP, triggering a series of proinflammatory responses [55–57]. CD39 degrades secreted ATP to AMP, and CD73 degrades AMP to adenosine, a molecule capable of suppressing immune cell activity and inflammation [23]. CD73 is a glycosylphosphatidylinositol-linked membrane protein that mediates the extracellular dephosphorylation of AMP to adenosine [19,20]. Previous studies have shown that CD73 is a potent suppressor of immune responses and that cells that express higher levels of CD39 and CD73 act to suppress inflammatory responses [10,11]. CD73-deficient mice exhibit stronger anti-tumor T cell responses [22], mice that overexpress CD73 exhibit significantly inhibited adaptive tumor immunosurveillance [58]. The role of CD73 in autoimmune diseases has also been examined. CD73 expression and adenosine generation have been associated with immune suppression in several diseases [58–62], and CD73 expression by CD4^+^CD25^+^Foxp3^+^ T cells contributes to their regulatory function [63–66]. CD73^-/-^ mice are resistant to induction of experimental autoimmune encephalomyelitis (EAE), even though CD4 T cells from CD73^-/-^ mice secrete more proinflammatory cytokines than WT mice and are able to induce EAE when transferred into naïve CD73^-/-^ T cell-deficient recipients [67].

In this study, we found that γδ T cells became activated and expressed decreased amounts of CD73 during the pre-clinical stage of EAU, which, in turn, increased the ability of γδ T cells to promote Th17 responses. The notion that CD73 expressed by γδ T cells modulates the autoimmune response was supported by two observations. First, a comparison of CD73-deficient (CD73^-/-^) and WT B6 (CD73^+/+^) mice showed that failure to express CD73 greatly reduced both the enhancing and suppressive activities of γδT cells. Second, examining whether differently activated γδ T cells are functionally different, we found that cytokine-exposed γδ T cells remained highly CD73 positive, whereas DC-exposed γδ T cells became CD73 negative. In the presence of exogenously added AMP, the cytokine-activated γδ T cells had a suppressive effect on αβ T cell activation, whereas DC-activated γδ T cells did not, suggesting that different levels of CD73 expression by γδ T cells regulate their enhancing and inhibiting activity. Thus, ATP/adenosine metabolism plays an important role in the interconversion of the enhancing and suppressive effects of γδ T cells, and CD73 expressed by γδ T cells plays an important role in this process.

The ecto-enzyme CD73 (ecto-5'-nucleotidase), a molecule pivotally involved in converting non-immunosuppressive AMP into immunosuppressive adenosine [20,21], is expressed by many cell types, including Treg cells [22,63,64,66], B cells [68] and endothelial cells [69]. Previous studies [23,38,70,71] have shown that CD73 molecules expressed on non-hematopoietic cells are functionally distinct from those expressed on hematopoietic cells. CD73 on hematopoietic cells suppresses T cell function, whereas CD73 on nonhematopoietic cells primarily affects T cell homing. Our study provided more information on the functional diversity of CD73 by showing that CD73 molecules expressed on γδ T cells were more effective at converting AMP to adenosine than those on other immune cells examined. The molecular mechanisms underlying this difference remain unclear. We found that there is no difference in the size and sequence of CD73 mRNA in γδ and αβ T cells (data not shown), and further studies are therefore needed to explain the functional difference.

Our present study showed that CD73^-/-^ mice are relatively resistant to actively induced EAU but not adoptively induced EAU (data not shown). It is likely that in adoptive transfer the “inducing phase” of disease is not involved, whereas in the induced system both the “inducing phase” and the “effector phase” are involved. Our results indicate that decreased disease susceptibility is due to changes in the “inducing phase” and not in the “effector phase” of CD73^-/-^ mice. The observations that γδ T cells from CD73^-/-^ mice possess enhanced Th17-promoting activity, whereas CD73^-/-^ mice are less susceptible to induction of EAU, suggest that CD73 molecules expressed on different cell types have diverse biological functions and that functional deficiency of CD73 on some cells promotes susceptibility to autoimmune disease, while deficiency on other cell types might promote suppression. CD73 is known to play an important role in various inflammation/pathogenesis-related events, including the penetration of the pathogenic T cells into the diseased organs [23,38,67], and in the control of vascular permeability [69,72,73]. Conceivably, the net effect of systemic CD73 deficiency may be determined by the sum of the enhancing and inhibitory effects on different pathogenic events; and in this case, selective targeting of CD73 function on specific cells should be more likely to achieve specific therapeutic goals. Further studies are apparently required for a better understanding of the biological functions of CD73. In this study we showed a new function of CD73: namely, CD73 shapes the regulatory effect of γδ T cells.

## Supporting Information

S1 ARRIVE Checklist The ARRIVE guidelines checklist. (PDF)
