# Correction: CD73 Expressed on γδ T Cells Shapes Their Regulatory Effect in Experimental Autoimmune Uveitis

**DOI:** 10.1371/journal.pone.0164502

**Published:** 2016-10-04

**Authors:** Dongchun Liang, Aijun Zuo, Ronglan Zhao, Hui Shao, Willi K. Born, Rebecca L. O'Brien, Henry J. Kaplan, Deming Sun

The following information is missing from the Funding section: This work was supported by an unrestricted grant from Research to Prevent Blindness (RPB), Inc., New York, New York.
